# Prevalence and correlates of depressive symptoms among Rohingya (forcibly displaced Myanmar nationals or FDMNs) older adults in Bangladesh amid the COVID-19 pandemic

**DOI:** 10.1017/gmh.2021.24

**Published:** 2021-06-14

**Authors:** Sabuj Kanti Mistry, A. R. M. Mehrab Ali, Nafis Md. Irfan, Uday Narayan Yadav, Rumana Ferdousi Siddique, Prince Peprah, Sompa Reza, Ziaur Rahman, Lisa Casanelia, Cathy O'Callaghan

**Affiliations:** 1 ARCED Foundation, 13/1 Pallabi, Mirpur-12, Dhaka, Bangladesh; 2 Centre for Primary Health Care and Equity, University of New South Wales, Sydney, Australia; 3 BRAC James P Grant School of Public Health, BRAC University, 68 Shahid Tajuddin Ahmed Sharani, Mohakhali, Dhaka-1212, Bangladesh; 4 Innovations for Poverty Action, New Haven, CT, USA; 5 Institute of Nutrition and Food Science, University of Dhaka, Dhaka 1000, Bangladesh; 6 Interdisciplinary Graduate Program in Human Toxicology, University of Iowa, Iowa, USA; 7 Department of Psychology, University of Dhaka, Dhaka 1000, Bangladesh; 8 Society for Health Extension and Development (SHED), Cox's Bazar, Bangladesh; 9 Torrens University, Melbourne, Australia

**Keywords:** COVID-19, depressive symptoms, forcibly displaced Myanmar nationals, GDS-15, Rohingya

## Abstract

**Background:**

Depression is globally a crucial communal psychiatric disorder, which is more common in older adults. The situation is considerably worse among millions of older (forcibly displaced Myanmar nationals or FDMNs) Rohingya adults, and the coronavirus disease-2019 (COVID-19) pandemic may exacerbate the already existing precarious situation. The present study investigated depressive symptoms and their associated factors in older adult Rohingya FDMNs in Cox Bazar, Bangladesh, during the COVID-19 pandemic.

**Method:**

A total of 416 older adults aged 60 years and above residing in Rohingya camps situated in the South Eastern part of Bangladesh were interviewed using a 15-item Geriatric Depression Scale (GDS-15) in Bengali language. Chi-square test was performed to compare the prevalence of depressive symptoms within different categories of a variable and a binary logistic regression model was performed to determine the factors associated with depressive symptoms.

**Results:**

More than 41% of Rohingya older adults had depressive symptoms (DS). Socio-demographic and economic factors such as living alone, dependency on family for living, poor memory, feelings of being left out, difficulty in getting medicine and routine medical care during COVID-19, perception that older adults are at highest risk of COVID-19 and pre-existing non-communicable chronic conditions were found to be significantly associated with developing DS. Higher DS was also evident among older female Rohingya FDMNs.

**Conclusion:**

DS are highly prevalent in older Rohingya FDMNs during COVID-19. The findings of the present study call for immediate arrangement of mental health care services and highlight policy implications to ensure the well-being of older FDMNs.

## Background

Depression is globally recognized as a public health problem, which is more common in older adults (Allan *et al*., 2014; Cheruvu and Chiyaka, 2019*a*). Data from the Global Burden of Diseases and the World Health Organization indicates approximately 264 million people aged 60 years and above have depressive symptoms (James *et al*., 2018; WHO, 2020*b*). For those over 65 years of age, depressive symptoms affects 10–16% of the global population and are largely determined by chronic illness, reduced functional abilities, cognitive impairment, and social isolation (WHO, 2017). Depression prevalence rates among older adults in low- and middle-income countries (LMICs), compared to high-income countries, are disparately high, yet this has received little attention in many LMICs, including Bangladesh (Patel, 2007).

There is concern about the health and wellbeing needs faced by older adults who are affected by conflict, natural disaster and displacement. Evidence has shown higher prevalence rates of mental health disorders including depression with refugee populations more than the general population (Cummings *et al*., 2011; Li, 2016).The health of this population who have chronic co-morbidities may be exacerbated by lack of access to treatment and support, isolation, distress and uncertainty of daily existence (Singh *et al*., 2018*a*) and migratory grief (Cummings *et al*., 2011). Evidence have documented that mental health disorders are quite common among forcefully displaced people around the world (Singh *et al*., 2018*a*; Abu Suhaiban *et al*., 2019; Khan and Haque, 2021). A systematic review conducted focusing mental health disorders among displaced people has revealed depression and anxiety as the second prevalent mental health problem after post-traumatic stress disorder (PTSD) (Morina *et al*., 2018). A longitudinal study from Germany conducted among Syrian refugee participants showed that 27% of the participants had clinical depression (Georgiadou *et al*., 2018).

The Rohingyas (forcibly displaced Myanmar nationals or FDMNs) are mostly a Muslim minority people residing in the Rakhine State of Myanmar, and have faced discrimination, violence and genocide for decades forcing them to leave their motherland and relocate in refugee camps in Bangladesh (UNHCR, 2018). The Rohingya camps are over-populated with an average population density of about 40 000 people per square kilometre (Kamal *et al*., 2020). People living in these camps lack access to clean water, sanitation and health services (Limon *et al*., 2020). Moreover, recent evidence suggests a high prevalence of non-communicable diseases (NCDs), particularly hypertension (51.5%) and diabetes (14.2%) (WHO, 2018; Alam *et al*., 2019; Joarder *et al*., 2020). Consequently, Rohingya FDMNs are at increased risk of coronavirus disease-2019 (COVID-19) (Islam and Yunus, 2020). Previous research also pointed that older adults from refugee camps are more vulnerable to COVID-19 and associated morbidities including mental disorders, particularly due to the poor socio-economic and physical conditions (Peprah, 2020). Post-traumatic exposure along with overwhelming COVID-19 threat, ongoing risks in their life, perceived fear because of uncertainties, pre-existing conditions and poor socio-economic status can result into depressive disorders among the displaced population. Existing evidence (Mehra *et al*., 2020; Sepúlveda-Loyola *et al*., 2020) also suggests that older adults are more likely to experience depressive disorders during the overwhelming situation such as COVID-19 and can be more exaggerated if older adults have difficult life circumstances such as being refugees, have pre-existing conditions and inadequate support mechanism required for sense of safety and overall life satisfaction.

The COVID-19 pandemic has appeared to be the most serious public health disaster around the globe. As of 30 November 2020, there were more than 62 million confirmed cases with nearly 1.5 million deaths worldwide which is increased above than 131 million and 2.8 million, respectively, by 6 April 2021. Similarly, by 30 November 2020, there were more than 462,000 confirmed COVID-19 cases with 6609 deaths in Bangladesh which is increased above 644,000 and 9318, respectively, by 6 April 2021 (WHO, 2020*a*). Whilst the entire global population is at risk of COVID-19, evidence suggests that certain population groups are more vulnerable than others. Older adults are particularly vulnerable to COVID-19 with increased risk of morbidity and mortality (CDC Covid- Response Team, 2020). Evidence suggests that, more than half (53%) of the COVID-19 deaths occurred among the people aged 60 years and above in India (The Times of India, 2020), which was 39% in Bangladesh (Rahman *et al*., 2020).

Historically, an increased prevalence of mental health conditions has been associated with the outbreak of pandemics. For example, during the H1N1 influenza virus outbreak in 2009, anxiety among the United Kingdom's general population increased by 10–30% (Rubin *et al*., 2010). Similarly, during the severe acute respiratory syndrome (SARS) epidemic, psychiatric morbidities, depression, and stress disorders increased (Sim *et al*., 2010). Furthermore, during the Ebola outbreak, in 2013–2016 in Guinea, Liberia, and Sierra Leone, the psychosocial well-being of the people was seriously hampered (Van Bortel *et al*., 2016). Likewise, evidence suggests that stress, anxiety, depressive symptoms, insomnia, denial, anger, and fear are associated with the COVID-19 (Torales *et al*., 2020). Also, the unplanned lockdown and subsequent difficulty in accessing food, health care, medication, and psychological support as well as social distancing and isolation could have exacerbated anxiety and depression in this particular population group (Cheruvu and Chiyaka, 2019*b*; Wu, 2020). For older people, a lack of financial support, family and social support, fear or anxiety over access to resources and emergency services have been attributed to the worsening of mental health conditions (Lee *et al*., 2020).

### Study context

Although several studies have been undertaken in the past to study mental health issues among older adults in Bangladesh, no study has explored depressive symptoms among older Rohingya FDMNs in Bangladesh during the COVID-19 pandemic. Therefore, the present study was undertaken to: (1) identify depressive symptoms among the Rohingya older FDMNs; and (2) investigate the factors associated with depressive symptoms. The findings of the study will inform the policy makers and practitioners of the countries that host displaced populations or refugees to explore the mental health issues among the older adults during any overwhelming situation like COVID-19.

## Methods

### Study design and participants

**Fig. 1.**
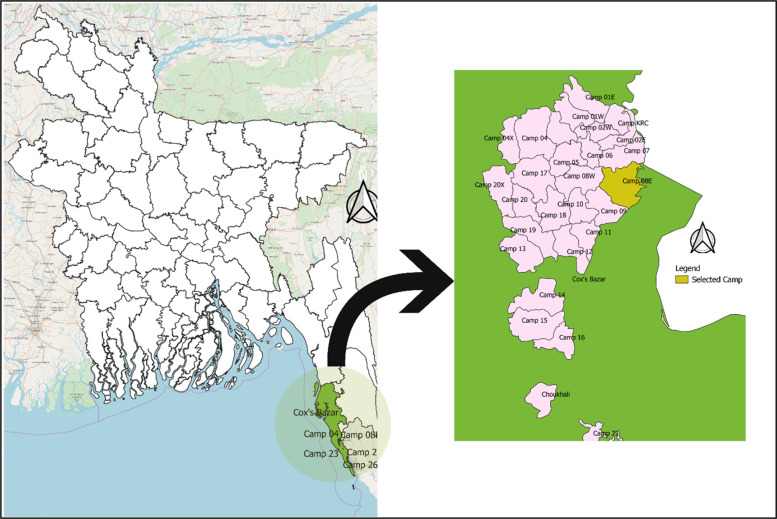
Geographical location of the study area

The study followed a cross-sectional design and was carried out in October 2020 among 416 older adults aged 60 years and older residing in Rohingya refugee camps situated in the South-Eastern part of Bangladesh. The sample size of 460 was calculated with the following assumptions: (unknown) prevalence of COVID-19-related misconceptions = 50%, sampling error = 5%, confidence interval = 95% and non-response rate = 20%. Of the 457 eligible participants approached, a total of 416 Rohingya older adults responded to the study (response rate 91%). There is a total of 34 Rohingya camps located in Cox's Bazar district from which Camp 08E (SSID CXB-210), located at *Ukhia* sub-district, was conveniently selected (Fig. 1). In the absence of the list of the older adults in Rohingya camps, a convenience sampling technique was used to identify the eligible participants in the selected camp. The data collectors continued visiting the households until the desired sample size was achieved. If the approached household did not have an eligible participant, the surveyors moved to the next one, and if the household had more than one older adult, all of them were interviewed. The inclusion criteria included age ⩾60 years and FDMN status. The exclusion criteria included severe mental illnesses (clinically proved schizophrenia, bipolar mood disorder, dementia/cognitive impairment), a hearing disability, or unable to communicate. We did not use any instrument to assess the exclusion criteria, instead the family members of the prospective participants were asked if the participant had severe mental illness or hearing disability.

### Measures

#### Outcome measure

The primary outcome of the study was depressive symptoms, measured using the 15-item Geriatric Depression Scale (GDS-15), a widely used scale for measuring depressive symptoms among older adults in both clinical and community settings (Yesavage *et al*., 1982). Briefly, each item in the scale is measured by yes/no questions. After reverse coding of some negatively worded items, a cumulative score of the 15-items, with ranges between 0 and 15, is calculated. The total score was dichotomized into probable absence (score ⩽ 5) or presence of depressive symptoms (>5) (El-Gilany *et al*., 2018). The GDS-15 scale has previously been used among Bangladeshi older adults (Disu *et al*., 2019; Rahman *et al*., 2019). We also found it to be a reliable scale, indicated by the high internal consistency (Cronbach's alpha 0.82) among our study participants.

#### Explanatory variables

Explanatory variables considered in this study were age (categorized as 60–69, 70–79, and ⩾80), sex (male/female), marital status (married/widowed), literacy (illiterate/literate), family size (⩽4 or >4), living arrangements (living alone or with family), dependence on family for a living (yes/no), walking distance to the nearest health center (<30 min/⩾30 min), memory or concentration problems (no problem/had memory or concentration problem), presence of pre-existing non-communicable chronic conditions (yes/no), feeling lack of companionship (hardly, sometimes/often), feelings of being left out (hardly, sometimes/often), concerned about COVID-19 (hardly, sometimes/often), overwhelmed by COVID-19 (hardly, sometimes/often), difficulty in getting food, medicine, and routine medical care during COVID-19 (no/yes), perception that older adults are at highest risk of COVID-19, and source of COVID-19-related information (radio, television, health workers, friends/family/neighbors).

Self-reported information on pre-existing medical conditions, such as arthritis, hypertension, heart diseases, stroke, hypercholesterolemia, diabetes, chronic respiratory diseases, chronic kidney disease, and cancer were collected. This information was verified with available health records (if available and/or with family members). We did not collect information on the income and occupation as the refugee population is mostly unemployed and dependent on social support.

### Data collection tools and techniques

A pre-tested semi-structured questionnaire developed in English and translated in Bengali was used to collect the information through face-to-face interview. Data were collected electronically using a SurveyCTO mobile app (https://www.surveycto.com/) by locally recruited data collectors who had previous experience administering health surveys in an electronic platform and who understood Rohingya dialects. The data collectors were trained extensively before the data collection using the Zoom platform.

The English version of the questionnaire was first translated into Bengali language and then back translated into English by two researchers to ensure content's consistency. The questionnaire was then piloted among a small sample (*n* = 10) of Rohingya refugee older adults to refine the language in the final version. However, the translated tool did not receive any corrections/suggestions from the participants. The pre-test results were not included in the final study.

### Statistical analysis

The distribution of the variables was assessed through descriptive analysis. The Chi-square test was performed to compare the prevalence of depressive symptoms within different categories of a variable with a 5% level of significance. We used a binary logistic regression model to determine the factors associated with depressive symptoms. The final model includes only the variables having *P* < 0.25 in the unadjusted analysis (Liang and Zeger, 1986). Both unadjusted and adjusted odds ratios (ORs) were reported with a 95% confidence interval (95% CI). All analyses were performed using the statistical software package Stata (Version 14.0).

## Results

### Participants' characteristics

The majority of the Rohingya FDMNs older adults were in the age range of 60–69 years of age (74%), male (60%) and married (93.5%). Around 60% of respondents belonged to a family of greater than four and literacy rates were found to be only 2.4%. Around 13% of refugee older adults reported living alone while 54% reported they depend on family for living. The major source of COVID-19-related information was through health workers followed by information from friends/family members and neighbors. The role of television in obtaining COVID-19 information was deemed to be very low (only 6%) (see Table 1).

**Table 1.** Sociodemographic and lifestyle characteristics of the respondents (*N* = 416)

Characteristics	*n*	%
Age (year, %)		
60–69	308	74.0
70–79	83	20.0
> = 80	25	6.0
Sex		
Male	251	60.3
Female	165	39.7
Marital status		
Married	389	93.5
Widow/Widower	27	6.5
Family size		
0–4	167	40.1
>4	249	59.9
Literacy		
Illiterate	406	97.6
Literate	10	2.4
Living arrangement		
Living with other family members	362	87.0
Living alone	54	13.0
Dependent on family for living		
No	191	45.9
Yes	225	54.1
Source of COVID-19-related information^a^		
Television	27	6.5
Radio	172	41.7
Health workers	298	72.2
Friends/family/neighbors	267	64.7

a Multiple responses.

### Prevalence of depressive symptoms

Overall, 41.6% of older Rohingya FDMNs had depressive symptoms (DS) according to the Geriatric Depression Scale (GDS-15). Bivariate analysis revealed that DS were found to be highest in 60–69 years old, higher in males and those who live in big families (family size >4) (Table 2). Again, DS was found to be statistically significantly higher in the living alone group and those who were not dependent on family for living. Similarly, higher DS percentages have been reported in groups who have higher walking distance time (>30 min) to a health care center and who have problems with memory or concentration.

**Table 2.** Participants' characteristics and bivariate analysis (*N* = 416)

Characteristics	Have DS *n* (%)	No DS *n* (%)	*p value*
Overall	173 (41.6)	243 (58.4)	
Age (year, %)			
60–69	133 (43.2)	175 (56.8)	0.172
70–79	34 (41.0)	49 (59.0)	
> = 80	6 (24.0)	19 (76.0)	
Sex			
Male	97 (38.6)	154 (61.4)	0.133
Female	76 (46.1)	89 (53.9)	
Family size			
0–4	61 (36.5)	106 (63.5)	0.086
>4	112 (45.0)	137 (55.0)	
Living arrangement			
Living with other family members	134 (37.0)	228 (63.0)	<0.001
Living alone	39 (72.2)	15 (27.8)	
Dependent on family for living			
No	104 (54.4)	87 (45.6)	<0.001
Yes	69 (30.7)	156 (69.3)	
Walking distance to the nearest health center			
<30 min	123 (37.3)	207 (62.7)	<0.001
⩾30 min	50 (58.1)	36 (41.9)	
Problem in memory or concentration			
No problem	131 (36.8)	225 (63.2)	<0.001
Had memory or concentration problem	42 (70.0)	18 (30.0)	
Feeling of lack of companionship			
Hardly	104 (34.7)	196 (65.3)	<0.001
Sometimes/often	69 (59.5)	47 (40.5)	
Feeling of left out			
Hardly	86 (31.4)	188 (68.6)	<0.001
Sometimes/often	87 (61.3)	55 (38.7)	
Difficulty obtaining food during COVID-19			
No difficulty	83 (31.4)	181 (68.6)	<0.001
Faced difficulty	82 (65.1)	44 (34.9)	
Difficulty getting medicine during COVID-19			
No difficulty	66 (23.8)	211 (76.2)	<0.001
Faced difficulty	105 (87.5)	15 (12.5)	
Difficulty receiving routine medical care during COVID			
No difficulty	72 (26.2)	203 (73.8)	<0.001
Faced difficulty	93 (78.8)	25 (21.2)	
Perceived that older adults at highest risk of COVID			
No	31 (20.5)	120 (79.5)	<0.001
Yes	142 (53.6)	123 (46.4)	
Pre-existing non-communicable chronic conditions			
No	92 (31.2)	203 (68.8)	<0.001
Yes	81 (66.9)	40 (33.1)	

Participants were asked whether they had feelings of a ‘lack of companionship’ or being ‘left out’ and if so, how often. Those who had either of these feelings and frequently (sometimes-often) suffered had almost twice as many DS as compared to those who hardly or rarely felt them. Similarly, DS were significantly higher among those people who had difficulty in obtaining food, medicine and routine medical care. Again, DS was observed to be two times higher in Rohingya FDMNs older adults who had other chronic diseases as compared to those who had none.

### Factors associated with depressive symptoms

The full model included the factors deemed to be associated with depressive symptoms (Table 2). The final model, based on the lowest AIC, retained the variables shown in Table 3. Hence, the model is adjusted for all the variables in Table 3. In the unadjusted model, living arrangements, family dependency for living, walking distance from health center, problems in memory/concentration, feeling a lack of companionship and being left out, difficulty in getting food, medicine and regular medical treatment during COVID-19, perception that older adults are at higher risk of COVID and suffering from a chronic or any other disease currently – were identified as significant risk factors for developing DS in older Rohingya refugee adults. However, after adjustment, except walking distance from a health center, feeling a lack of companionship and difficulty in getting food − all other factors remained significant for DS (Table 3).

**Table 3.** Factors associated with depressive symptoms among participants (*N* = 416)

	Crude			Adjusted		
	OR	*p value*	95% CI	OR	*p value*	95% CI
Age (year, %)						
60–69	1.00			1.00		
70–79	0.91	0.717	0.56–1.49	1.36	0.738	0.22–8.40
> = 80	0.42	0.069	0.16–1.07	1.62	0.621	0.24-10.90
Gender							
Male	1.00			1.00		
Female	1.36	0.134	0.91–2.02	1.09	0.817	0.52–2.27
Family size						
0–4	1.00			1.00		
>4	1.42	0.087	0.95–2.12	1.23	0.565	0.60–2.53
Living arrangement						
Living with other family members	1.00			1.00		
Living alone	4.42	<0.001	2.35–8.33	4.58	0.005	1.58–13.30
Dependent on family for living						
Yes	1.00			1.00		
No	2.70	<0.001	1.81–4.04	3.35	0.001	1.61–6.95
Walking distance from health center						
<30 min	1.00			1.00		
⩾30 min	2.34	0.001	1.44–3.79	1.63	0.301	0.65-4.11
Problem in memory or concentration						
No problem	1.00			1.00		
Had memory or concentration problem	4.01	<0.001	2.22–7.25	5.05	0.002	1.83–13.93
Feeling lack of companionship						
Hardly	1.09			1.00		
Sometimes/often	2.77	<0.001	1.78–4.30	1.41	0.384	0.65–3.06
Feeling of being left out						
Hardly	1.00			1.00		
Sometimes/often	3.46	<0.001	2.26–5.28	2.96	0.005	1.38–6.37
Difficulty getting food during COVID-19						
No difficulty	1.00			1.00		
Faced difficulty	4.06	<0.001	2.59–6.37	1.92	0.118	0.85–4.36
Difficulty getting medicine during COVID-19						
No difficulty	1.00			1.00		
Faced difficulty	22.38	<0.001	12.19–41.09	7.25	<0.001	2.93–17.96
Difficulty receiving routine medical care during COVID-19						
No difficulty	1.00			1.00		
Faced difficulty	10.49	<0.001	6.25–17.59	3.09	0.015	1.24–7.68
Perceived that older adults are at highest risk of COVID-19						
No	1.00			1.00		
Yes	4.47	<0.001	2.81–7.10	5.55	<0.001	2.57–11.97
Pre-existing non-communicable chronic conditions						
No	1.00			1.00		
Yes	4.47	<0.001	2.84–7.02	5.88	<0.001	2.68–12.93

It was found that older adult Rohingya refugees who were living alone had almost four and half times greater odds of developing DS as compared to those who were living with family (aOR 4.58, CI 1.58–13.30). Older adult refugees with low incidence of memory and concentration problems had five times higher odds of suffering from DS as compared to those who had no memory problems (aOR 5.05, CI 1.83–13.93). Again, those who had feelings of being left out (sometimes or very often) were three times more likely to develop DS as compared to those who hardly had this feeling (aOR 2.96, CI 1.38–6.37). Similarly, people who faced difficulty in getting food during the COVID-19 crisis and those who faced difficulty in getting medicine, had almost two times higher odds (aOR 1.92, CI 0.85–4.36) and more than 7 times greater odds (aOR 7.25, CI 2.93–17.96), respectively, of suffering from DS as compared to older adult refugees who had not been facing any difficulty obtaining food and medicine. On the other hand, those who perceived older adults to be of highest risk of contracting COVID-19 had more than five times higher odds (aOR 5.55, CI 2.57–11.97) of developing DS as compared to those who did not have this perception. Lastly, we found older adult refugees who were suffering from any other chronic disease had almost 6 times higher risk of developing DS as compared to those who had no chronic disease (aOR 5.88, CI 2.68–12.93).

## Discussion

The present study reports DS and their association among Rohingya FDMNs older adults in a refugee camp setting in Cox bazar, Bangladesh during the COVID-19 pandemic. The current study is unique as it is the first to assess the level of DS and their associations among the older Rohingya FDMNs in Bangladesh. The study's findings might guide the relevant stakeholders, policy makers and international agencies to design approaches to address this unmet mental health need for this ultra-vulnerable population.

This investigation revealed that almost 42% of the participants had DS. Globally, evidence of different scales of mental health and depression suggest that prevalence rates of a major depressive disorder (MDD), generalized anxiety disorder (GAD), or PTSD are 44, 40, and 36% in refugees settings (Turrini *et al*., 2017). Similarly, a previous study on Rohingya FDMNs adults in Bangladesh reported prevalence of MDD, PTSD, and GAD to be 89, 36, and 14%, respectively (Riley *et al*., 2017), but this study was done in adults and before the COVID-19 pandemic era. This signifies that even compared to the same scale of depression (GAD), the vulnerability of developing DS was higher in older Rohingya FDMNs in the COVID-19 era although studies from different groups, times and instrumental settings should be taken into consideration while interpreting and comparing the results.

Our study found higher DS in older adults female Rohingya FDMNs as compared to their male counterparts and this corroborates with previous findings in Rohingya FDMNs in Bangladesh (Turrini *et al*., 2017), Rohingya FDMNs in Malaysia (Kaur *et al*., 2020), Syrian refugees in Iraq (Mahmood *et al*., 2019) and Bosnian refugees in Sweden (Blight *et al*., 2006). It is evident from both local and global evidence that refugees have disproportionate outcomes based on gender and females suffer more psychosocial trauma, indicating the influence of both psychosocial and biological factors. Women are two times more susceptible than men to develop DS irrespective of nations, cultures, and ethnicities (Weissman *et al*., 1996; Mayo Clinic, 2019). Since women have been perceived to be in a lower social position to men in terms of power and status, they often encounter more traumas and inequities (poverty, harassment, disrespect, and forced choices). Even, exposure to the same stressors make women more susceptible to develop depression than men because of their reported greater sensitivity to stress hormones, constraints in using more adaptive coping strategies, and tendency to provide negative evaluation to emergencies than men (Verma *et al*., 2011). Women are also at greater risk of developing stress-related symptoms as they have more caregiving responsibilities and are expected to balance work and household tasks in all types of situations which contributes to the development of depression in women. Being a member of refugee community in the pandemic might exacerbate this situation for older women.

Furthermore, we found living alone, lack of companionship and feelings of being left out were significant factors associated with DS. Undoubtedly older refugees not only have inherent vulnerability to stress or depression, dependency on others for food, shelter, medicine, and treatment, all of which might contribute to increased DS. Other study show correlations between DS and age, stressors such as migratory grief and death of spouse, and coping/social support variables (Cummings *et al*., 2011). In addition, the emergence of COVID-19 has added more stress in relation to feelings of fear and anxiety in older adult refugees regarding access to the essentials. It is important therefore for policy makers to offer especial psycho-social support packages for Rohingya FDMNs older adults and engage community frontline health workers to promote a sense of self-esteem, inclusion and integration in the community.

Similarly, difficulty in getting food, medicine and routine medical care during COVID-19 have been identified as being significant risk factors for developing DS in older Rohingya FDMNs. A lack of access to food and food insecurity has been linked to psychological stress in previous studies conducted with Rohingya FDMNs in Bangladesh, Malaysia (Turrini *et al*., 2017; Kaur *et al*., 2020) and among refugees and displaced people in other settings such as South Africa, Sri Lanka and Canada (Hamid and Musa, 2010; Siriwardhana *et al*., 2013; Maharaj *et al*., 2017). A recent review also identified poor nutrition, lack of access to shelter, health care, public services, and safety as risk factors to psychiatric illness and coronavirus in refugee settings (Júnior *et al*., 2020). Therefore, current ongoing food aid and medical services should continue to be provided uninterrupted with safe social distancing.

Interestingly, older FDMNs who perceived that older adults are at a higher risk of contracting COVID-19 and those who did not depend on their family members for living had higher risk of experiencing DS. This is important to consider and address when providing psychological support to those with mental health conditions. A well informed and realistic outlook on COVID-19 susceptibility is necessary − since it provides the person with accurate knowledge, however, this increased fear and anxiety may lead them to more and higher level of DS. Therefore, it is crucial to consider the possible negative effect of the message while communicating and providing psycho-social support to this group. This is a variable that more easily controlled as opposed to their social situation or the presence of physical ailments. Another important finding − that single older adults are at more risk of DS as compared to those who depend on family members calls for further attention to support those older FDMNs who live alone, where family members or volunteers could play a pivotal role.

In our study, we found older Rohingya FDMNs suffering from pre-existing non-communicable chronic conditions were six times more likely to develop DS. In research with older Kurdish refugees in the United States, the strongest predictor of depressive symptoms was the number of medical conditions (Cummings *et al*., 2011). Similar findings were also observed in other studies that report chronic disease to be associated with higher stress, anxiety and depression in older adults during COVID-19 although this study was carried out among the general older population (Gorrochategi *et al*., 2020). Chronic conditions are often associated with change in several biological processes including changes in the redox state, inflammatory markers, and immunity system that makes the neurological system more prone to stress and trauma (Chapman *et al*., 2005). Therefore, older FDMNs with pre-existing non-communicable chronic conditions need utmost attention considering their vulnerability to COVID-19 and depression. In addition, an effective communication strategy should be developed so that knowing the facts − about communicable diseases makes more case-fatality and detrimental outcomes in older people less common and does not in it itself become a message creating worry and anxiety to that group.

The present study outcome provides evidence for the need to develop and deliver psychosocial support policies and packages for older Rohingya FDMNs. This study is novel from several benchmarks. It provides quantitative insights into mental health and its associated factors for older Rohingya FDMNs older adults in Bangladesh during COVID-19 pandemic for the first time. Secondly, this refugee setting is unique since Rohingya refugee camps in Cox bazar, Bangladesh are the largest and most concentrated refugee camps in the world. The results from this study indicate, Rohingya FDMNs in Bangladesh have high DS after the outbreak of COVID-19 and have several psycho-social support issues (such as feelings of dependency, being left out and loneliness), infrastructure issues (walking distance of health center), supply issues (inadequate access to food and medicine during COVID-19) and pre-existing non-communicable chronic conditions; all of which should be taken into consideration for targeting psychological or mental health intervention programs for older Rohingya FDMNs (especially women) in Bangladesh.

This study is novel from several benchmarks. It provides quantitative insights into mental health and its associated factors for older Rohingya FDMNs older adults in Bangladesh during COVID-19 pandemic for the first time. Secondly, this refugee setting is unique since Rohingya refugee camps in Cox bazar, Bangladesh are the largest and most concentrated refugee camps in the world. Despite the uniqueness and timeliness of our study, the study does have some limitation. First, as we conveniently selected a camp and purposively selected the participants from the camp there is possibility of selection bias. The purposive sampling procedure also limits the generalizability of the study for the entire camp population. Secondly, while some of the previous studies used GDS-15 to measure depressive symptoms among older adults in Bangladesh, the tool was not validated among the Bangladeshi population. Third, the study was cross sectional in nature; so, causality cannot be checked and established. Fourth, considering the fact that regression analysis is not the choice of method for a non-probability sample, one should be cautious of interpreting the results from the regression analysis. Yet, we have tried to address the bias that might have resulted through convenience sampling by adding the sociodemographic variables that can possibly result in bias in the regression model. Finally, a mixed method study with a relatively larger sample size could have had better insights into the associated factors of DS.

### Implications for policy, practice and further research

The present study provides findings which have significant implications for policy, practice and further research. The results from this study indicate need of community-based program aims to screen, diagnose and manage DS (Yadav *et al*., 2020) through psychosocial support packages for older Rohingya FDMNs. Additionally, the findings from this study might guide the relevant stakeholders, policy makers and international agencies to design psychological first aid module to address this unmet mental health need for this ultra-vulnerable population. Our findings also suggest the need of similar studies focusing displaced older adults in various international setting where resettlement of refugees and displaced population have taken place.. The finding further suggests the need of mental health support for the older adults who are residing in several refugee or displaced people at various international settings during any overwhelming condition such as COVID-19 pandemic.

## Conclusion

COVID-19 is contributing vigorously to the occurrence of mental health conditions like depressive symptoms among older adults. The present study revealed that the prevalence of DS is very high among older Rohingya FDMNs who are already in the position of increased susceptibility due to their age and other circumstances. Several crucial factors gave rise to this high prevalence that need to be addressed by the policy makers as well as community frontline health workers to reach an equilibrium for this population. Some new perspectives are also revealed that highlight the need for mental health intervention programs. In conclusion, the present study demonstrates the prerequisites of basic needs along with psycho-social support to be provided to ensure the well-being of older Rohingya FDMNs. Moreover, based on our findings we emphasize the need of early screening and management of DS among this target population which may prevent worsening of the symptoms, contributing to improved quality of life.

## References

[ref1] Abu Suhaiban H, Grasser LR and Javanbakht A (2019) Mental health of refugees and torture survivors: a critical review of prevalence, predictors, and integrated care. International Journal of Environmental Research and Public Health 16, 2309. 10.3390/ijerph16132309 PMC6651013 31261840

[ref2] Alam N, Kenny B, Maguire J, Mcewen S, Sheel M and Tolosa M (2019) Field epidemiology in action: an Australian perspective of epidemic response to the Rohingya health emergencies in Cox's Bazar, Bangladesh. Global Biosecurity 1, 1–4.

[ref3] Allan CE, Valkanova V and Ebmeier KP (2014) Depression in older people is underdiagnosed. The Practitioner 258, 19–22. 25065018

[ref4] Blight KJ, Ekblad S, Persson J-O and Ekberg J (2006) Mental health, employment and gender. Cross-sectional evidence in a sample of refugees from Bosnia-Herzegovina living in two Swedish regions. Social Science & Medicine 62, 1697–1709. 16171914 10.1016/j.socscimed.2005.08.019

[ref5] CDC Covid- Response Team (2020) Severe outcomes among patients with coronavirus disease 2019 (COVID-19) – United States, February 12−March 16, 2020. MMWR. Morbidity and Mortality Weekly Report 69, 343–346. 32214079 10.15585/mmwr.mm6912e2 PMC7725513

[ref6] Chapman DP, Perry GS and Strine TW (2005) Peer reviewed: the vital link between chronic disease and depressive disorders. Preventing Chronic Disease 2, 1–10. PMC1323317 15670467

[ref7] Cheruvu VK and Chiyaka ET (2019 *a*) Prevalence of depressive symptoms among older adults who reported medical cost as a barrier to seeking health care: findings from a nationally representative sample. BMC Geriatrics 19, 1–10. 31319807 10.1186/s12877-019-1203-2 PMC6639933

[ref8] Cheruvu VK and Chiyaka ET (2019 *b*) Prevalence of depressive symptoms among older adults who reported medical cost as a barrier to seeking health care: findings from a nationally representative sample. BMC Geriatrics 19, 192. 31319807 10.1186/s12877-019-1203-2 PMC6639933

[ref9] Cummings S, Sull L, Davis C and Worley N (2011) Correlates of depression among older Kurdish refugees. Social Work 56, 159–168. 21553579 10.1093/sw/56.2.159

[ref10] Disu TR, Anne NJ, Griffiths MD and Mamun MA (2019) Risk factors of geriatric depression among elderly Bangladeshi people: a pilot interview study. Asian Journal of Psychiatry 44, 163–169. 31382211 10.1016/j.ajp.2019.07.050

[ref11] El-Gilany A-H, Elkhawaga GO and Sarraf BB (2018) Depression and its associated factors among elderly: a community-based study in Egypt. Archives of Gerontology and Geriatrics 77, 103–107. 29734054 10.1016/j.archger.2018.04.011

[ref12] Georgiadou E, Zbidat A, Schmitt GM and Erim Y (2018) Prevalence of mental distress among Syrian refugees with residence permission in Germany: a registry-based study. Frontiers in Psychiatry 9, 393. 30210373 10.3389/fpsyt.2018.00393 PMC6121182

[ref13] Gorrochategi MP, Munitis AE, Santamaria MD and Etxebarria NO (2020) Stress, anxiety, and depression in people aged over 60 in the COVID-19 outbreak in a sample collected in Northern Spain. The American Journal of Geriatric Psychiatry 28, 993–998. 32576424 10.1016/j.jagp.2020.05.022 PMC7261426

[ref14] Hamid AA and Musa SA (2010) Mental health problems among internally displaced persons in Darfur. International Journal of Psychology 45, 278–285. 22044013 10.1080/00207591003692620

[ref15] Islam MM and Yunus MDY (2020) Rohingya refugees at high risk of COVID-19 in Bangladesh. The Lancet Global Health 8, e993–e994. 32593313 10.1016/S2214-109X(20)30282-5 PMC7316456

[ref16] James SL, Abate D, Abate KH, Abay SM, Abbafati C, Abbasi N, Abbastabar H, Abd-Allah F, Abdela J and Abdelalim A (2018) Global, regional, and national incidence, prevalence, and years lived with disability for 354 diseases and injuries for 195 countries and territories, 1990–2017: a systematic analysis for the Global Burden of Disease Study 2017. The Lancet 392, 1789–1858. 10.1016/S0140-6736(18)32279-7 PMC6227754 30496104

[ref17] Joarder T, Sutradhar I, Hasan MI and Bulbul MMI (2020) A record review on the health Status of Rohingya refugees in Bangladesh. Cureus 12, 1–8. 10.7759/cureus.9753 PMC7489778 32944468

[ref18] Júnior JG, De Sales JP, Moreira MM, Pinheiro WR, Lima CKT and Neto MLR (2020) A crisis within the crisis: the mental health situation of refugees in the world during the 2019 coronavirus (2019-nCoV) outbreak. Psychiatry Research 288, 113000. 32353696 10.1016/j.psychres.2020.113000 PMC7156944

[ref19] Kamal A-H, Huda M, Dell C, Hossain S and Ahmed S (2020) Translational strategies to control and prevent spread of COVID-19 in the Rohiynga refugee camps in Bangladesh. Global Biosecurity 1, 1–10.

[ref20] Kaur K, Sulaiman AH, Yoon CK, Hashim AH, Kaur M, Hui KO, Sabki ZA, Francis B, Singh S and Gill JS (2020) Elucidating mental health disorders among Rohingya refugees: a Malaysian perspective. International Journal of Environmental Research and Public Health 17, 6730. 10.3390/ijerph17186730 PMC7559711 32942770

[ref21] Khan S and Haque S (2021) Trauma, mental health, and everyday functioning among Rohingya refugee people living in short- and long-term resettlements. Social Psychiatry and Psychiatric Epidemiology 56, 497–512. 33015727 10.1007/s00127-020-01962-1

[ref22] Lee SW, Yang JM, Moon SY, Yoo IK, Ha EK, Kim SY, Park UM, Choi S, Lee S-H and Ahn YM (2020) Association between mental illness and COVID-19 susceptibility and clinical outcomes in South Korea: a nationwide cohort study. The Lancet Psychiatry 7, 1025–1031. 32950066 10.1016/S2215-0366(20)30421-1 PMC7498216

[ref23] Li W (2016) Comparative study on social-economic status, trauma and mental health disorders among older and younger refugees in Australia. Journal of Tropical Psychology 6, 1–9.

[ref24] Liang K-Y and Zeger SL (1986) Longitudinal data analysis using generalized linear models. Biometrika 73, 13–22.

[ref25] Limon MTI, Jubayer MF, Ahmed MU, Rahman H and Kayshar MS (2020) Rohingya refugees and coronavirus disease-2019: addressing possible jeopardy from the perspective of Bangladesh. Asia Pacific Journal of Public Health 32, 529–530. 32749144 10.1177/1010539520947887

[ref26] Maharaj V, Tomita A, Thela L, Mhlongo M and Burns JK (2017) Food insecurity and risk of depression among refugees and immigrants in South Africa. Journal of Immigrant and Minority Health 19, 631–637. 26984226 10.1007/s10903-016-0370-x PMC5026864

[ref27] Mahmood HN, Ibrahim H, Goessmann K, Ismail AA and Neuner F (2019) Post-traumatic stress disorder and depression among Syrian refugees residing in the Kurdistan region of Iraq. Conflict and Health 13, 1–11. 31728157 10.1186/s13031-019-0238-5 PMC6842196

[ref28] Mayo Clinic (2019) Depression in women: Understanding the gender gap [Online]. Available at https://www.mayoclinic.org/diseases-conditions/depression/in-depth/depression/art-20047725#:~:text=After%20puberty%2C%20depression%20rates%20are,may%20continue%20throughout%20the%20lifespan (Accessed 07 December 2020).

[ref29] Mehra A, Rani S, Sahoo S, Parveen S, Singh AP, Chakrabarti S and Grover S (2020) A crisis for elderly with mental disorders: relapse of symptoms due to heightened anxiety due to COVID-19. Asian Journal of Psychiatry 51, 102114. 32334406 10.1016/j.ajp.2020.102114 PMC7166027

[ref30] Morina N, Akhtar A, Barth J and Schnyder U (2018) Psychiatric disorders in refugees and internally displaced persons after forced displacement: a systematic review. Frontiers in Psychiatry 9, 433. 30298022 10.3389/fpsyt.2018.00433 PMC6160546

[ref31] Patel V (2007) Mental health in low- and middle-income countries. British Medical Bulletin 81, 81–96. 17470476 10.1093/bmb/ldm010

[ref32] Peprah P (2020) Ageing out of place in COVID-19 pandemic era: how does the situation look like for older refugees in camps? Archives of Gerontology and Geriatrics 90, 1–3. 10.1016/j.archger.2020.104149 PMC7305710 32593091

[ref33] Rahman MS, Rahman MA and Rahman MS (2019) Prevalence and determinants of loneliness among older adults in Bangladesh. International Journal of Emerging Trends in Social Sciences 5, 57–64.

[ref34] Rahman MS, Afroze L and Rahman MS (2020) COVID-19 pandemic and older people in Bangladesh. Dr. Sulaiman Al Habib Medical Journal 2, 83–84.

[ref35] Riley A, Varner A, Ventevogel P, Taimur Hasan MM and Welton-Mitchell C (2017) Daily stressors, trauma exposure, and mental health among stateless Rohingya refugees in Bangladesh. Transcultural Psychiatry 54, 304–331. 28540768 10.1177/1363461517705571

[ref36] Rubin GJ, Potts HWW and Michie S (2010) The impact of communications about swine flu (influenza A H1N1v) on public responses to the outbreak: results from 36 national telephone surveys in the UK. Health Technology Assessment 14, 183–266. 20630124 10.3310/hta14340-03

[ref37] Sepúlveda-Loyola W, Rodríguez-Sánchez I, Pérez-Rodríguez P, Ganz F, Torralba R, Oliveira DV and Rodríguez-Mañas L (2020) Impact of social isolation due to COVID-19 on health in older people: mental and physical effects and recommendations. The Journal of Nutrition, Health & Aging 24, 938–947. 10.1007/s12603-020-1500-7 PMC7597423 33155618

[ref38] Sim K, Chan YH, Chong PN, Chua HC and Soon SW (2010) Psychosocial and coping responses within the community health care setting towards a national outbreak of an infectious disease. Journal of Psychosomatic Research 68, 195–202. 20105703 10.1016/j.jpsychores.2009.04.004 PMC7094450

[ref39] Singh N, Bassb J, Sumbadzec N, Rebokb G, Perrina P, Paichadzea N and Robinsona C (2018) Identifying mental health problems and idioms of distress among older adult internally displaced persons in Georgia. Social Science & Medicine, 211, 39–47. 29886407 10.1016/j.socscimed.2018.05.007

[ref40] Siriwardhana C, Adikari A, Pannala G, Siribaddana S, Abas M, Sumathipala A and Stewart R (2013) Prolonged internal displacement and common mental disorders in Sri Lanka: the COMRAID study. PloS One 8, e64742. 23717656 10.1371/journal.pone.0064742 PMC3661540

[ref41] The Times of India (2020) Covid toll in working age group at 10% now. Available at http://timesofindia.indiatimes.com/articleshow/80018558.cms?utm_source = contentofinterest&utm_medium=text&utm_campaign=cppst [Online]. Available at https://timesofindia.indiatimes.com/india/covid-toll-in-working-age-group-at-11-now/articleshow/80018558.cms (Accessed 08 April 2021).

[ref42] Torales J, O'higgins M, Castaldelli-Maia JM and Ventriglio A (2020) The outbreak of COVID-19 coronavirus and its impact on global mental health. International Journal of Social Psychiatry 66, 317–320. 10.1177/0020764020915212 32233719

[ref43] Turrini G, Purgato M, Ballette F, Nosè M, Ostuzzi G and Barbui C (2017) Common mental disorders in asylum seekers and refugees: umbrella review of prevalence and intervention studies. International Journal of Mental Health Systems 11, 51. 28855963 10.1186/s13033-017-0156-0 PMC5571637

[ref44] UNHCR (2018) Rohingya Refugee Crisis [Online]. Available at https://www.unocha.org/rohingya-refugee-crisis (Accessed 07 December 2020).

[ref45] Van Bortel T, Basnayake A, Wurie F, Jambai M, Koroma AS, Muana AT, Hann K, Eaton J, Martin S and Nellums LB (2016) Psychosocial effects of an Ebola outbreak at individual, community and international levels. Bulletin of the World Health Organization 94, 210. 26966332 10.2471/BLT.15.158543 PMC4773931

[ref46] Verma R, Balhara YPS and Gupta CS (2011) Gender differences in stress response: role of developmental and biological determinants. Industrial Psychiatry Journal 20, 4. 22969173 10.4103/0972-6748.98407 PMC3425245

[ref47] Weissman MM, Bland RC, Canino GJ, Faravelli C, Greenwald S, Hwu H-G, Joyce PR, Karam EG, Lee C-K and Lellouch J (1996) Cross-national epidemiology of major depression and bipolar disorder. JAMA 276, 293–299. 8656541

[ref48] WHO (2017) Evidence profile: depressive symptoms [Online]. Available at https://www.who.int/ageing/health-systems/icope/evidence-centre/ICOPE-evidence-profile-depressive.pdf?ua=1 (Accessed 23 March 2021).

[ref49] WHO (2018) Bangladesh: Rohingya refugee crisis 2017–2018 [Online]. Available at http://www.searo.who.int/mediacentre/emergencies/bangladesh-myanmar/public-healthsituation-analysis-may-2018.pdf (Accessed).

[ref50] WHO (2020 *a*) WHO coronavirus disease (COVID-19) dashboard [Online]. Available at https://covid19.who.int/ (Accessed 07 December 2020).

[ref51] WHO (2020 *b*) The World Health Report 2001: mental disorders affect one in four people [Online]. Available at https://www.who.int/news/item/28-09-2001-the-world-health-report-2001-mental-disorders-affect-one-in-four-people (Accessed 03 November 2020).

[ref52] Wu B (2020) Social isolation and loneliness among older adults in the context of COVID-19: a global challenge. Global Health Research and Policy 5, 1–3. 32514427 10.1186/s41256-020-00154-3 PMC7272234

[ref53] Yadav UN, Thapa TB, Mistry SK, Pokhrel R and Harris MF (2020) Socio-demographic characteristics, lifestyle factors, multi-morbid conditions and depressive symptoms among Nepalese older adults. BMC Psychiatry 20, 1–9. 32456611 10.1186/s12888-020-02680-3 PMC7249669

[ref54] Yesavage JA, Brink TL, Rose TL, Lum O, Huang V, Adey M and Leirer VO (1982) Development and validation of a geriatric depression screening scale: a preliminary report. Journal of Psychiatric Research 17, 37–49. 7183759 10.1016/0022-3956(82)90033-4

